# Factors associated with adherence to preventive measures against COVID-19, among adult Bahraini residents

**DOI:** 10.1038/s41598-024-76843-7

**Published:** 2024-11-03

**Authors:** Adoub Al Derazi, Mariam Bubshait, Ameena Albinjasim, Amina Al Binzayed, Hessa Bugahoos, Noor Al Rumaihi, Noora Abuzeyad, Noora Al Sadeh, Noora Al Khaja, Zainab Salmeen, Tawfeeq Naseeb, Noor Al Shenawi

**Affiliations:** 1 College of Medicine and Medical Sciences, Arabian Gulf University, Manama, Bahrain; 2 Department of Family and Community Medicine, Arabian Gulf University, Manama, Bahrain

**Keywords:** COVID-19_**1**_, Bahrain_**2**_, Preventive measures_**3**_, Mask use_**4**_, Social distance_**5**_, Hygiene_**6**_, Risk factors, Epidemiology

## Abstract

The COVID-19 pandemic has been a life-altering experience. It compelled individuals to alter their attitudes and habits and adopt measures to prevent the risks of infection. This research discusses factors that determine adherence to these measures, with the aim of enhancing compliance in future pandemics. A descriptive cross-sectional study was conducted using snowball non-random sampling through an online Qualtrics survey targeting Bahraini residents, aged 18 and older. The sample included 1,008 participants, and data were analyzed using SPSS, and presented in tables and graphs. The majority of the participants were Bahraini females with a bachelor’s degree, between the ages of 20–29 (43.2%). Awareness of COVID-19 transmission was high, with 87.7%. The adherence rates for preventive measures were high: mask usage at 98.8%, hygiene practices at 93.7%, and social distancing at 92.2%. Older adults aged 50 + showed the highest consistency in compliance (p = 0.001). Individuals with chronic diseases were more likely to adhere to mask use (p < 0.001). Conversely, those experiencing negative emotions about social distancing were more likely to visit crowded places (p = 0.031), indicating a psychological barrier. Mask use exhibited the highest adherence at 98.8%, while overall compliance was notably high among educated and retired individuals. Public health campaigns should target younger demographics with education about crowded spaces and address emotional barriers to social distancing. Future strategies can be tailored to promote adherence among diverse populations, enhancing community resilience against pandemics.

## Introduction

COVID-19 first appeared as an epidemic in Wuhan, China, and was later declared a pandemic by the World Health Organization in March 2020. Many countries all around the world implemented different preventive measures, such as hygiene, mask use, and social distancing to limit the spread of the virus^1^.

In February 2020, Bahrain witnessed the first COVID-19 case. Since then, it started putting down certain guidelines and measures to be followed. Three main preventive measures were implemented by the Ministry of Health for the public: hygiene, mask use, and social distancing. Staying at home was one of the first measures implemented in Bahrain on the 25th of February 2020. After cases started increasing gradually, masks were made compulsory for the whole community^2^.

A cross-sectional study conducted by Jose R et al. in India in 2020 aimed to understand human behavior and perception towards preventive measures through the Health Belief Model. The study found that when individuals living in Kerala were equipped with adequate knowledge of COVID-19, they adhered to the preventive measures set by the government through their perceived susceptibility and severity^3^. Health Belief Model focuses on many components, such as risk of acquiring a disease, seriousness of the condition, effectiveness of taking an action to reduce risk, and perceived obstacles to perform a recommended health action. In this case the factor that will trigger individuals to adhere and take action is the outbreak of COVID-19 in their region.

Studies around face mask use revealed that individuals are more likely to adhere if they believe it’s for their protection. In particular, Asian countries complied more with mask use than Western countries. Many individuals claimed that mask use is inconvenient and that other preventive measures are more effective. Studies with countries that implemented legislation to mask use showed the highest compliance^4–6^.

Social distancing in literature showed a strong relationship between adherence and emotional state, the desire to protect oneself and others is what motivated people to isolate themselves. Yet economic factors played a major role in acting as a barrier to compliance with these preventive measures^7–9^.

Research showed that the main driving cause of hygiene compliance is daily habit, and its biggest challenge is access to clean water. Alcohol-based sanitizers were considered a good alternative to protect against the spread of the COVID-19 pandemic with no perceived adverse effects^10–12^. Studies published in the Arabian Gulf region in 2022 stated that compliance with preventive measures was greatly associated with the public’s general knowledge regarding the health situation^13^.

All in all, the previously discussed studies are limited in the fact that none of them focused on cues of action as an incentive to adhere to the preventive measures. Therefore, to elevate the standard of this study in comparison to previous ones this will be explored.

Our study aim is to increase awareness about the factors which lead to adherence to preventative measures (mask use, hand washing, and social distancing) against COVID–19, among adult Bahraini residents (18 years and above). The objectives of this paper are to examine and analyze factors associated with adherence to the preventive measures against COVID-19 in adult Bahraini residents during 2021. Furthermore, we aim to provide data regarding people’s personal beliefs, perceptions, and knowledge regarding COVID-19 in adult Bahraini residents during 2021. We hypothesize that in this global pandemic, fear dictated adherence to following preventive measures, especially since the virus was highly transmissible and infectious.

## Methods

The type of study implemented in this paper is a descriptive cross-sectional study. The study population included adult Bahraini residents 18 years and above with access to smart devices with social media platforms such as Twitter, Snapchat, Instagram, and WhatsApp during August 2021. Illiterate individuals and those unable to complete online surveys due to technology illiteracy, blindness, or lack of access to technology or social media were excluded. In addition, Bahrainis living abroad were not included in the study. The sampling technique integrated was snowball non-random sampling based on the number of Bahraini residents who came across the e-surveys (Qualtrics) and completed it. The minimum required sample size for the study was 384 participants to estimate an expected prevalence of population adherence to COVID-19 preventive measures of 50% with a margin of error of 5% at a confidence interval of 95% at the national level.

The lack of previous studies conducted in Bahrain on the prevalence of people’s adherence to preventive measures left us with no objective reference value to refer to for the value of P. Hence, a standard value of 0.5 was utilized to yield the largest sample size number.

### Data collection

Due to restrictions of the pandemic, data used in this study was collected by e-surveys (Qualtrics) and sent randomly on social media platforms such as WhatsApp, Twitter, Snapchat, and Instagram. The survey was sent alongside a two-minute video demonstrating the struggles faced by medical practitioners, aimed to encourage viewers to complete the survey. The video was filmed in Arabic as it’s the mother language of the Bahraini population.

The research was built upon a self-administered anonymous questionnaire in both English and Arabic. The questions were in a multiple-choice format (mainly close-ended questions), aimed to eliminate the subjectivity of results that accompany scale-based answers (strongly agree/disagree). Close-ended questions were efficiently used by multiple other researchers, especially in settings where participants provide answers remotely. The questionnaire included sociodemographic variables such as age, gender, education level, and occupation.

The questionnaire incorporated elements of the Health Belief Model such as perceived susceptibility, severity, barriers, benefits, and health motivation within the designated sections of the preventive measures. This will provide us with information regarding which preventive measures were/weren’t adhered to and why. Associations were to be made between the factors, as well as facilitating the analysis of which preventive measure is the most prominent and convenient. Questions used in the survey were derived from previous literature that studied preventive measures in infectious diseases, and some incorporated the Health Belief Model. Implementing questions from published literature strengthened the validity of these questions as measures of assessment^5,9,14^.

All methods were performed in accordance with the relevant guidelines and regulations.

### Statistical analysis

Statistical Packaging of Social Sciences (SPSS) 23 software was used for data entry and analysis. Frequencies and percentages were computed for categorical variables. Meanwhile, mean and standard deviation were computed for continuous variables. Bar charts were used to present categorical variables. Chi-squared test was used to determine whether there is a significant relationship between two categorical variables. Additionally, the McNemar test was utilized to compare two dichotomous dependent variables to determine the change in adherence to the preventive measures over time. For both statistical analyses, a P-value of less than 0.05 was considered statistically significant. Binary logistic progression was used to explore the risk factors that affect adherence to preventive measures (impact on adherence).

## Results

### Demographical relation to the adherence to preventive measures

**Fig. 1:**
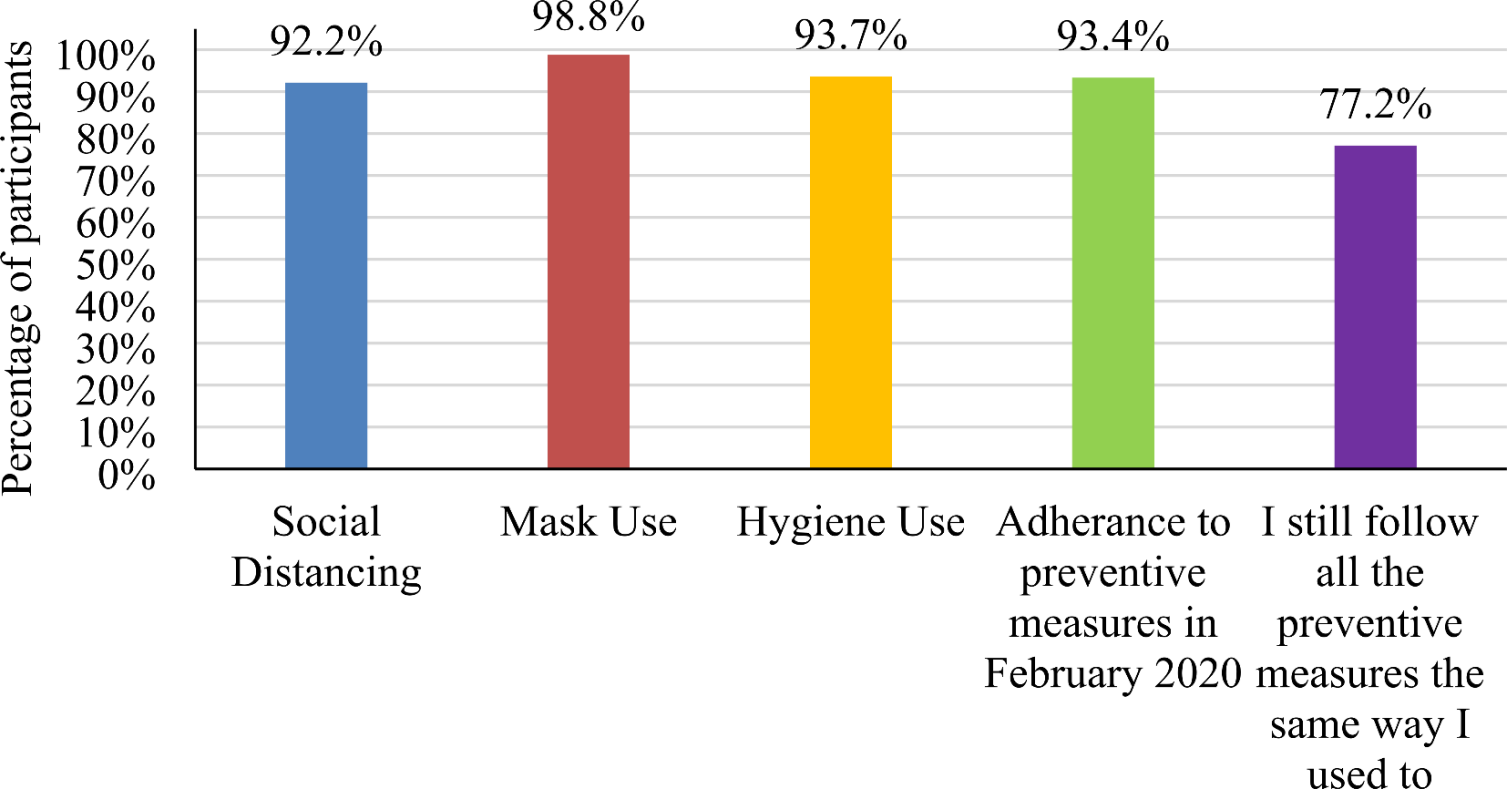
Percentage of adherence to social distancing, mask use, hygiene use, and changes in percentage of adherence.

Of the 1,008 participants, there were 435 in the age group 20–29 years, and 43.2% of them were Bahraini females with a university degree (Table 1). Most of the participants adhered to all the preventive measures (mask use, hygiene use, and social distancing), with mask use being the most adhered to and social distancing the least. (Fig. 1). There was an association between age (all ages) and adherence to social distancing (p: <0.001), mask use (p:0.017), and hygiene (0.005) (Table 2). Highly significant values were shown between employment status and social distancing (p:<0.001), as well as between employment status and hygiene (p:0.006) (Table 2).

**Table 1:** Demographic characteristics of participants (total = 1008).

	*n* (%)
**Age in years** ^**1**^	
18–19	114 (11.3)
20–29	435 (43.2)
30–39	162 (16.1)
40–49	131 (13)
≥ 50	166 (16.5)
**Nationality**	
Bahraini	805 (79.9)
Non-Bahraini	203 (20.1)
**Gender**	
Male	270 (26.8)
Female	738 (73.2)
**Education level**	
High school or below	361 (35.8)
University	497 (49.3)
Higher studies	150 (14.9)
**Employment status**	
Student	389 (38.6)
Employed	368 (36.5)
Unemployed	111 (11)
Retired	140 (13.9)
**Accommodation/living**	
Flat/apartment	209 (20.7)
Private House	602 (59.7)
Shared House	143 (14.2)
Collective accommodation	34 (3.4)
University accommodation	20 (2)
**Number of rooms** ^**2**^	
< 5	406 (40.3)
5–8	507 (50.3)
> 8	95 (9.4)
**Number of people in accommodation** ^**3**^	
< 5	288 (28.6)
5–7	503 (49.9)
> 7	217 (21.5)
**Chronic diseases**	
Asthma	83 (8.2)
Diabetes	62 (6.2)
Hypertension	80 (7.9)
Heart problems	13 (1.3)
Others	152 (15.1)

(1) Mean is 32.6 ± 13.9 years. (2) Mean is 5.3 ± 2.8 rooms. (3) Mean is 6.0 ± 3.2 people.

**Table 2:** Relationship between demographic characteristics and adherence to social distancing, mask use, and hygiene.

	Social Distancing	*P*-value	Mask Use	*P*-value	Hygiene Use	*P*-value
	*n* (%)		*n* (%)		*n* (%)	
**Age in years**						
18–19	99 (86.8)	< 0.001*	114 (100)	0.017*	100 (87.7)	0.005*
20–29	382 (87.8)		424 (97.5)		403 (92.6)	
30–39	156 (96.3)		161 (99.4)		152 (93.8)	
40–49	129 (98.5)		131 (100)		129 (98.5)	
≥ 50	163 (98.2)		166 (100)		160 (96.4)	
**Nationality**						
Bahraini	747 (92.8)	0.137	794 (98.6)	0.477	760 (94.4)	0.049*
Non-Bahraini	182 (89.7)		202 (99.5)		184 (90.6)	
**Gender**						
Male	248 (91.9)	0.824	267 (98.9)	1.000	257 (95.2)	0.227
Female	681 (92.3)		729 (98.8)		687 (93.1)	
**Education level**						
High school or below	333 (92.2)	0.025*	359 (99.4)	0.366	334 (92.5)	0.337
University	450 (90.5)		489 (98.4)		466 (93.8)	
Higher studies	146 (97.3)		148 (98.7)		144 (96)	
**Employment status**						
Student	340 (87.4)	< 0.001*	385 (99)	0.259	356 (91.5)	0.006*
Employed	349 (94.8)		363 (98.6)		355 (96.5)	
Unemployed	104 (93.7)		108 (97.3)		99 (89.2)	
Retired	136 (97.1)		140 (100)		134 (95.7)	
**Accommodation/living**						
Flat/apartment	191 (91.4)	0.972	208 (99.5)	0.057	198 (94.7)	0.098
Private House	556 (92.4)		597 (99.2)		569 (94.5)	
Shared House	132 (92.3)		138 (96.5)		128 (89.5)	
Collective accommodation	32 (94.1)		33 (97.1)		32 (94.1)	
University accommodation	18 (90)		20 (100)		17 (85)	

Note: P-values were computed by using Chi-square test. P-value < 0.05.

Table 3 indicates that adherence to social distancing was significantly influenced by age and educational level. Participants aged 30–39 were 5.361 times more likely to adhere to social distancing than those aged 18–19 with a 95% CI (1.608, 17.876). Those aged 40–49 were 21.529 times more likely to adhere to social distancing than those aged 18–19 with a 95% CI (2.898, 159.943) and participants ≥ 50 years were 35.092 times more likely to adhere to social distancing than those aged 18–19 with a 95% CI (3.381, 364.184). Also, there was a significant association between education level and social distancing (p:0.025) (Table 2). Participants with high school degrees or below were 1.821 times more likely to adhere to social distancing than those with university degrees with a 95% confidence interval (1.001, 3.311) (Table 3).

**Table 3:** Binary logistic regression between demographic characteristics and social distancing.

Characteristics	*P*-value	Adjusted Odd ratio	95% CI for odd ratio	
			Lower	Upper
**Age in years**				
18–19 (Reference)				
20–29	0.378	1.381	0.674	2.832
30–39	0.006*	5.361	1.608	17.876
40–49	0.003*	21.529	2.898	159.943
≥ 50	0.003*	35.092	3.381	364.184
**Nationality**				
Bahraini (Reference)				
Non-Bahraini	0.117	0.596	0.312	1.139
**Gender**				
Male (Reference)				
Female	0.117	1.572	0.893	2.765
**Education level**				
High school or below (Reference)				
University	0.049*	0.549	0.302	0.999
Higher studies	0.888	0.917	0.275	3.061
**Employment status**				
Student (Reference)				
Employed	0.592	1.225	0.583	2.572
Unemployed	0.616	1.259	0.511	3.102
Retired	0.190	0.237	0.027	2.042
**Accommodation/living**				
Flat/apartment (Reference)				
Private House	0.862	1.060	0.550	2.041
Shared House	0.517	1.323	0.567	3.086
Collective accommodation	0.971	1.033	0.186	5.738
University accommodation	0.500	1.729	0.352	8.484

Note: P-values were computed by using Chi-square test. P-value < 0.05.

Meanwhile, in reference to 18–19 year olds, participants aged 40–49 were 12.365 times more likely to adhere to hygiene and those aged 50 and above were 7.061 times more likely to adhere with 95% confidence intervals (2.003, 76.317) and (1.145,43.561) respectively. A relationship between nationality and hygiene use showed significance with a p-value of 0.049 (Table 2). Bahrainis were also 2.278 times more likely to have better hygiene with a 95% CI (1.136, 4.566).

### Impact of knowledge on adherence

Among the survey participants, 87.7% were fully aware of how the virus spreads. Most of the survey participants adhered to social distancing (92.2%, 929), mask use (98.8%, 996), and hygiene use (93.7%, 944). In addition, 37.6% of participants were worried about facing long-term effects, while 37.5% believed the virus is “just like the flu and it will go.” People were mainly concerned about getting admitted to the hospital or dying when being infected with COVID-19 (Table 4).

**Table 4:** Knowledge on COVID-19 preventive measures (total = 1008).

	*n* (%)
**I am aware of how the virus spreads**,** the reason for mask use**,** washing hands and social distancing**	
Yes, I am fully aware	884 (87.7)
Yes, I know the basics	122 (12.1)
No, I am not aware	2 (0.2)
**The transmission power of COVID-19 is high**	
Yes	945 (93.8)
No	63 (6.3)
**I think I am at risk of getting COVID-19**	
Yes	462 (45.8)
No	241 (23.9)
I don’t know	305 (30.3)
**The minimum distance I should maintain between myself and others to reduce the risk of spreading COVID-19 is**	
At least 0.5 m	33 (3.3)
At least 1 m	381 (37.8)
At least 2 m	577 (57.2)
I don’t know	17 (1.7)
**Reason for not following all the preventive measures the same way I used to**	
My adherence improved	79 (34.3)
I do not have the patience to follow preventive instructions	40 (17.4)
I do not care if I get COVID-19	8 (3.5)
I think COVID-19 is not real	3 (1.3)
I am tired of COVID-19 lifestyle	100 (43.5)
**If COVID-19 infected me**	
It is just like the flu; it will go	378 (37.5)
I am worried about-facing long-term effects (i.e. loss of smell & breathing problems)	379 (37.6)
I am concerned that I will get admitted to the hospital	179 (17.8)
I am concerned that I have a high chance of dying	72 (7.1)
**I got vaccinated**	
Yes	964 (95.6)
No	44 (4.4)
**Reason for vaccination**	
It reduces the risk of getting COVID-19	246 (25.5)
To reduce my adherence to the preventive measures	4 (0.4)
To protect myself and my family	553 (57.4)
To follow recommendations of Ministry of Health	85 (8.8)
To travel and go out	76 (7.9)
**Reason of not vaccinating**	
I applied to take the vaccine	14 (31.8)
I don’t need it	17 (38.6)
I think it harms me	13 (29.5)

### Factors associated with adherence

Results showed that 70.7% of participants always used sanitizers or washed their hands after touching a dirty surface, while 5.4% did not include sanitization in their routine. Some said this was due to the lack of washrooms, others were busy or sometimes forgot. Some participants were concerned about their skin as hand sanitizers cause dryness, itching, redness, and burning side effects. The majority of the participants, reaching 94.4% felt comfortable and safe after washing their hands which motivated 84.3% of them to keep washing their hands in the future (Table 5).

**Table 5:** Hygiene behavior among study participants (total = 1008).

	*n* (%)
**Frequency of using hand sanitizer/wash hands after touching a dirty surface**	
Always	713 (70.7)
Sometimes	215 (21.3)
I don’t use hand sanitizer/ hand wash	54 (5.4)
I use gloves	26 (2.6)
**Reason for not washing hands/sanitizing after touching a dirty surface**	
Lack of washrooms nearby	33 (61.1)
I am too busy/ lazy	9 (16.7)
I forget	5 (9.3)
It causes dryness in my hands	1 (1.9)
I don’t think its effective in protecting me from COVID 19	5 (9.3)
It has harmful side effects (itching, redness & burning)	1 (1.9)
**After washing my hands**,** I feel comfortable and safe**	
Yes	952 (94.4)
No	56 (5.6)
**This feeling motivates me to wash my hands in the future**	
Yes	803 (84.3)
No	149 (15.7)

The data showed that 69.5% of participants agree that mask use is mandatory, with a mean agreement of 7.6 ± 3.3. Restrictions on gatherings increased the adherence to social distancing as full lockdown was not implemented with a mean of 7.4 ± 2.9. Participants concerned about the health of their family were mostly adherent to social distancing with a mean of 9.3 ± 1.9 (Table 6).

**Table 6:** Reasons of wearing mask and reasons of adherence to social distancing (total = 1008).

	Mean ± SD^1^
**I choose to wear mask in public because**	
It is mandatory	7.6 ± 3.3
Avoid paying violation fee	4.9 ± 3.8
It protects my family and I from the virus	9.0 ± 2.3*
I am/a family member is a part of the high-risk group	7.1 ± 3.5
**Full lockdown was not implemented**,** which of the following increased your adherence to social distancing?**	
Restrictions on gatherings	7.4 ± 2.9
Work/ school from home	7.4 ± 3.1
Closing public spaces	7.1 ± 3.0
**I adhere to social distancing because**	
I don’t want to violate the rules	7.6 ± 3.2
I am concerned about my health	8.5 ± 2.6*
I am concerned about the health of my family	9.3 ± 1.9*

1. Mean ± SD was computed from a scale 1 to 10, where 1 is the lowest importance and 10 is the highest importance.

There is a strong association between having a chronic disease/s and adhering to mask use as one’s family member is a part of the high-risk group with a statistically significant p-value of < 0.001 (Table 7).

**Table 7:** Relationship between chronic disease and adherence to mask use because a family member is part of a high-risk group.

Chronic Disease	Adherence to mask use because a family member is part of a high-risk group		*P*-value
	Yes	No	
	*n* (%)	*n* (%)	
Yes	218 (73.6)	78 (26.4)	< 0.001*
No	411 (57.7)	301 (42.3)	

Note: P-values were computed by using Chi-square test. P-value < 0.05.

### Change in adherence over time

A noticeable difference was seen in the level of compliance of individuals at the beginning of the pandemic (February 2020) and at the time of completing the survey 18 months later (August 2021) with a 16.2% reduction (Fig. 1). This is reinforced by significant p-value < 0.001(Table 8).

**Table 8:** Change over time in adherence to preventive measures.

February 2020		August 2021		*P*-value
Yes	No	Yes	No	
*n* (%)	*n* (%)	*n* (%)	*n* (%)	
941 (93.4)	67 (6.6)	778 (77.2)	230 (22.8)	< 0.001*

Note: P-values were computed by using McNemar test. P-value < 0.05.

It was noticed that there was a significant decrease in the number of young people still adhering to the preventive measures since the beginning of the pandemic, from 89.5% adherence to 59.6% only (Table 9). In reference to participants aged 18–19, those aged 50 and above were 11.082 times more likely to adhere the same way they used to with a confidence interval of 95% (3.952, 31.078), making them the most likely to maintain same levels of adherence compared to the rest of the age groups. They are followed by those aged 40–49 who were 6.361 times more likely with a 95% CI (2.718, 14.887). Then, participants aged 30–39 were 4.061 times more likely to keep adhering than 18–19 year olds with a confidence interval of 95% (1.966, 8.386). Finally, those aged 20–29 were 1.696 times more likely to adhere the same way with a confidence interval of 95% (1.037, 2.774) (Table 10).

**Table 9:** Relationship between demographic characteristics and adherence to preventive measures.

	Adherence to preventive measures in February 2020		*P*-value	I still follow all the preventive measures the same way I used to		*P*-value
	Yes	No		Yes	No	
	*n* (%)	*n* (%)		*n* (%)	*n* (%)	
**Age in years**						
18–19	102 (89.5)	12 (10.5)	0.440	68 (59.6)	46 (40.4)	< 0.001*
20–29	407 (93.6)	28 (6.4)		305 (70.1)	130 (29.9)	
30–39	152 (93.8)	10 (6.2)		134 (82.7)	28 (17.3)	
40–49	125 (95.4)	6 (4.6)		117 (89.3)	14 (10.7)	
≥ 50	155 (93.4)	11 (6.6)		154 (92.8)	12 (7.2)	
**Nationality**						
Bahraini	759 (94.3)	46 (5.7)	0.018*	626 (77.8)	179 (22.2)	0.381
Non-Bahraini	182 (89.7)	21 (10.3)		152 (74.9)	51 (25.1)	
**Gender**						
Male	253 (93.7)	17 (6.3)	0.787	212 (78.5)	58 (21.5)	0.541
Female	688 (93.2)	50 (6.8)		566 (76.7)	172 (23.3)	
**Education level**						
High school or below	334 (92.5)	27 (7.5)	0.727	268 (74.2)	93 (25.8)	0.008*
University	466 (93.8)	31 (6.2)		380 (76.5)	117 (23.5)	
Higher studies	141 (94)	9 (6)		130 (86.7)	20 (13.3)	
**Employment status**						
Student	360 (92.5)	29 (7.5)	0.347	265 (68.1)	124 (31.9)	< 0.001*
Employed	342 (92.9)	26 (7.1)		296 (80.4)	72 (19.6)	
Unemployed	108 (97.3)	3 (2.7)		89 (80.2)	22 (19.8)	
Retired	131 (93.6)	9 (6.4)		128 (91.4)	12 (8.6)	
**Accommodation/living**						
Flat/apartment	201 (96.2)	8 (3.8)	0.248	172 (82.3)	37 (17.7)	< 0.001*
Private House	558 (92.7)	44 (7.3)		469 (77.9)	133 (22.1)	
Shared House	133 (93)	10 (7)		96 (67.1)	47 (32.9)	
Collective accommodation	32 (94.1)	2 (5.9)		33 (97.1)	1 (2.9)	
University accommodation	17 (85)	3 (15)		8 (40)	12 (60)	

Note: P-values were computed by using Chi-square test. P-value < 0.05.

**Table 10:** Binary logistic regression between demographic characteristics and still follow all the preventive measures the same way I used to.

Characteristics	*P*-value	Adjusted Odd ratio	95% CI for odd ratio	
			Lower	Upper
**Age in years**				
18–19 (Reference)				
20–29	0.035*	1.696	1.037	2.774
30–39	< 0.001*	4.061	1.966	8.386
40–49	< 0.001*	6.361	2.718	14.887
≥ 50	< 0.001*	11.082	3.952	31.078
**Nationality**				
Bahraini (Reference)				
Non-Bahraini	0.040*	0.618	0.391	0.977
**Gender**				
Male (Reference)				
Female	0.140	1.333	0.910	1.951
**Education level**				
High school or below (Reference)				
University	0.848	0.962	0.651	1.424
Higher studies	0.445	1.282	0.678	2.426
**Employment status**				
Student (Reference)				
Employed	0.093	0.644	0.386	1.076
Unemployed	0.766	0.913	0.503	1.658
Retired	0.450	0.681	0.251	1.848
**Accommodation/living**				
Flat/apartment (Reference)				
Private House	0.022*	0.575	0.357	0.924
Shared House	0.002*	0.420	0.241	0.732
Collective accommodation	0.038*	9.061	1.136	72.292
University accommodation	0.003*	0.213	0.077	0.589

Note: P-values were computed by using Chi-square test. P-value < 0.05.

Bahrainis were more adherent to preventive measures than non-Bahrainis with a p-value of 0.018 (Table 9). This is further supported by Table 11 which shows that Bahraini participants were 3.45 times more likely to adhere to preventive measures than Non-Bahrainis with a 95% confidence interval (1.7,6.9) in February 2020. Meanwhile, in August 2021, Bahraini participants were 1.618 times more likely to maintain adherence with a 95% confidence interval (1.023, 2.557) and this is significant with a p-value of 0.040 (Table 10).

**Table 11:** Binary logistic regression between demographic characteristics and adherence to preventive measures in February 2020.

Characteristics	*P*-value	Adjusted Odd ratio	95% CI for odd ratio	
			Lower	Upper
**Age in years**				
18–19 (Reference)				
20–29	0.210	1.698	0.742	3.890
30–39	0.200	2.216	0.656	7.478
40–49	0.072	3.479	0.896	13.500
≥ 50	0.221	2.425	0.587	10.019
**Nationality**				
Bahraini (Reference)				
Non-Bahraini	< 0.001*	0.290	0.145	0.579
**Gender**				
Male (Reference)				
Female	0.867	0.948	0.505	1.777
**Education level**				
High school or below (Reference)				
University	0.450	1.293	0.663	2.522
Higher studies	0.564	1.322	0.512	3.415
**Employment status**				
Student (Reference)				
Employed	0.089	0.456	0.184	1.128
Unemployed	0.527	1.538	0.405	5.838
Retired	0.318	0.492	0.122	1.980
**Accommodation/living**				
Flat/apartment (Reference)				
Private House	0.004*	0.259	0.103	0.654
Shared House	0.021*	0.284	0.098	0.827
Collective accommodation	0.828	1.205	0.224	6.476
University accommodation	0.115	0.299	0.067	1.342

Note: P-values were computed by using Chi-square test. P-value < 0.05.

Also, retired participants and those with higher studies sustained their compliance from the beginning of the pandemic (93.6%, 131). Thus, there is an association between educational level and adherence to preventive measures.

With regard to accommodation, all participants adhered equally at the beginning of the pandemic. However, after some time, a significant drop in levels of adherence was noted amongst all except those who lived in collective accommodation, with a p-value of < 0.001. Participants who lived in flats/apartments had relatively unchanged adherence. On the other hand, most of those who lived in university accommodations stopped adhering as much (from 85 to 40%) (Table 9). Looking further using the binary regression model in Table 11, in February 2020, participants who lived in flats/apartments were 3.86 times more likely to adhere than those who lived in private houses with a 95% CI (1.53, 9.71). Flat/apartment residents were also 3.52 times more likely to adhere than participants who lived in shared houses with a 95% CI (1.21, 10.20). In August 2021, those who lived in flats/apartments were 4.695 times more likely to maintain adherence than participants who lived in university accommodation with a 95% CI (1.697, 12.987). They were also 2.381 times more likely to maintain adherence than those who lived in shared houses and 1.739 times more likely to maintain adherence than those who lived in private houses with a 95% confidence interval (1.366, 4.149) and (1.082, 2.801) respectively. Conversely, those who lived in collective accommodation were 9.061 time more likely to adhere than those in flats/apartments with a 95% CI (1.136, 72.292).

## Discussion

This study aimed to determine people’s adherence to preventive measures against COVID-19. The research emphasizes three core preventive measures, which were heavily complied by the participants, including social distancing (92.2%), mask use (98.8%), and hygiene use (93.7%). A similar study conducted in 2021 in Spain by Beca-Martínez MT et al. reported a slightly lower level of compliance with each of the preventive measures with scores of 84.7% social distancing, 91.6% mask use, and 90.1% hygiene use. Of all three, mask use has the highest level of compliance, which can also be seen in our results^15^. The high level of compliance seen in our result stems from the awareness and knowledge of the participants (87.7%) towards the virus and its transmission. A recent study in Kerla further supports this idea. It demonstrates higher rates of behavioral changes regarding the preventive measures, which were observed in 93.8% of the participants, who were more aware of COVID-19^3^. When applying the Health Belief Model (HBM) to these results, the knowledge and awareness the participants had contributed to their “Perceived Benefit” to following the preventive measures. This benefit ultimately stemmed from their “Perceived Susceptibility” to aquire COVID-19 after being educated about it. A study from Saudi Arabia stated that compliance with preventive measures showed a reduction in disease mortality and morbidity^16^.

In this study, it is highlighted that a significant number (94.3%) of Bahraini participants were adherent and strictly followed the preventive measures throughout the pandemic. The motive behind this may be due to patriotism and the internal sense of devotion and attachment that Bahraini participants had towards their country. Bahrainis might have felt responsible for achieving safety and welfare on a national level. Hence, according to the HBM acting as a “Cue to action” to following these measures. Meanwhile, in terms of adherence to the preventive measures among the participants, all age groups adhered relatively similarly with an insignificant p-value. This result can be related to the beginning of the pandemic, as people were unaware of the novel virus, its complications, and its effects. However, it was noted that with the progression of the pandemic, this p-value became significant. Participants above the age of 50 years old maintained their adherence in contrast to younger age groups. A possible reason can be related to the elderly’s perception of being considered a part of a high-risk group, as well as the responsibility they feel toward their family. This demonstrates the importance of “Perceived susceptibility” and “Perceived severity” as driving factors for compliance. In contrast, younger individuals don’t hold as many responsibilities as adults and can be easily influenced by their friends. This could also stem from the impatience and boredom that grew in individuals over time, thus influencing their behavior. This phenomenon can be observed in a study by Wolff W et al. which showed that individuals high in boredom found compliance to preventive measures to be more difficult, which decreased their adherence^17^. In contrast to these results, a study in Italy on Age-related differences in the perception of COVID-19 showed no significant age-related differences in compliance with preventive measures^18^.

As for the association between educational level and compliance to preventive measures, the data collected displayed that participants with higher studies (86.7%) and retired (91.4%) sustained their compliance from February 2020 till the time of data collection. A notion to this result can be because people with higher studies, and those retired are more aware of the consequences and might have a wider knowledge regarding infectious diseases, more specifically COVID-19. Hence, their “Perceived Benefit” of actually adhering to prevent acquiring the disease. Further supporting this data, 97.3% of people with higher studies complied with social distancing, and (97.1%) of retired participants adhered to social distancing. However, participants with university degrees had the least compliance to social distancing. This is also reflected in the adherence seen in participants in university accommodations who were the least likely to maintain adherence 18 months into the pandemic. A similar study conducted in Mozambique in 2021 found that being older, and more educated was correlated with a higher level of compliance^19^. A study done in Poland showed that individuals with higher educational levels showed higher levels of adherence to preventive measures compared to others^20^.

Individual differences in perception towards COVID-19 were demonstrated through the results obtained, in which (37.5%) viewed the virus “just like the flu; it will go” and the others (37.6%) were “worried about facing long-term effects”. These differences might originate from word of mouth and the wide range of experiences of COVID-19 symptoms. Especially since some people faced severe complications, and some had milder symptoms. In contrast, a similar study in India by Roy found that only 43% of participants perceived the virus as highly contagious^21^. These results further emphasize “Perceived Susceptibility” as a compelling factor to adherence.

Adherence to mask use was the highest among the preventive measures with a score of 98.8%. In comparison to the literature review by Bricker, western countries adhered to mask use with < 35%, and Eastern Asian countries adhered with 75%. According to Bricker, this adherence is attributed to each individual’s aim to protect themselves from the virus^4^. In this research, when asked about the reason for people’s adherence to mask use, the majority answered “concern and protection towards themselves and their families” with a mean of 9.0 ± 2.3. However, the higher value seen in Bahrainis could be tied back to mask use being the only preventative measure in Bahrain. Mask use was enforced on citizens as people were fined and prevented access to services if they did not comply. Despite this, a study conducted through the Macau analysis where mask use was not implemented with legislation shows high adherence with 96.4%. However, this may be due to mask use being a pre-existing social norm^6^. Overall, this elucidates the importance of legislation for the implementation of mask use as a preventive measure in cultures with no predisposing habits toward it. When utilizing the HBM, they serve as an effective “Cue to Action”.

Adherence to hygiene in Bahraini residents is 92.2%. In fact, from a wider perspective comparing our data to a study by Gibson JM published in May 2020, which establishes a positive relation between hygiene and reflective motivation with a retention percentage of 69.4%. Similar findings were seen in our research data, 94.4% of the participants felt psychologically comfortable and safe after washing their hands, and (84.3%) of participants felt motivated to wash their hands in the future. Thus, supporting the theory of reflective motivation regarding Gibson’s study^10^. In contrast to research conducted by Mohammed SG in 2020 with 87% adherence, which revealed that the main barriers to hand washing practices were work overload followed by shortage of water and soap^11^. Our results reflect that barriers to hygiene use in Bahrain are not an economic issue like other countries. This is further highlighted by another similar result demonstrating a positive correlation in the practice of hygiene use by Bahraini citizens, with Bahrainis (94.4%) and non-Bahrainis (90.6%), as Bahrainis were also 2.278 times more likely to have better hygiene with a 95% CI (1.136, 4.566). A possible factor playing a key role is that Bahrain is a developing country, with access to hygiene products and facilities, which can promote an easy approach to their citizens. Overall, demonstrating that when a “Perceived Barrier” is reduced compliance increases.

Social distancing is adhered to by 92.2% of the total participants. However, age groups 40 and above that have higher education, and are retired adhered to most. Social distancing being the least adhered to in Bahraini residents, may be a consequence of the cultural importance of family and friend gatherings. Hence, familial decisions dictate whether an individual will adhere to regulations or not. As they act as a Perceived Barrier by the Bahraini population leading to them having reduced “Self-efficacy” when applying the HBM. This is supported by findings by Coroiu et al. in North America and Europe, with a range of 80–89% found for adherence to social distancing. Especially as 84% of their participants felt a sense of responsibility to protect their community^9^. A study in France said that people who perceive other individuals as trustworthy ‘family members’ are more likely to not follow precautions since they have a sense that they will not get the infection from those trustworthy individuals^22^.

Furthermore, the accommodation type reflected that people who lived in a collective accommodation inclined their adherence by 3%. Conversely, a drop of 13–40% was noticed among the rest. This shows that people living together might have feared the consequences of sharing parts of their living space, which made them more adherent to guidelines. On the other hand, regression of adherence was noted among university students. This might stem from the fact that strict regulations might have led to boredom among students, which was not to their advantage. Moreover, university students, especially of younger age, might underestimate the importance of adhering to preventive measures. People living in their apartments showed unchanged adherence since they are living in their comfort zone with no implicated fear.

### Limitations

An important limitation in our research is the method of sampling “snowball method’’ which resulted in a selection bias. Most of us researchers were females, thus the survey was majorly distributed to female participants. Cultural factors play an important role here, especially in our communities where social relationships are more favorable within the same gender. We tried to adjust for this bias by employing male relatives and friends as initial participants to distribute the survey online.

Along with the distributed e-survey, an attached video was sent to emphasize the aim of the study and encourage individuals of various backgrounds and varying socioeconomic levels to participate in the research. Since the majority of the Bahraini population speaks Arabic, we chose to narrate the video in Arabic. This fact might have minimized responses from non-Arabic speaking participants. Follow up studies including a more diverse population and different methods of sampling are needed to enhance the generalizability of the findings, however replicating this study may be challenging due to the unique circumstances during which it was conducted. Nevertheless, the findings of this study help us to understand adherence to preventive measures whilst facing an unforeseen global health emergency.

Our study population was more adherent to hand washing more than any other preventative measure for COVID-19 because it is part of our religious and cultural beliefs. On average people living in Bahrain wash their hands at least five times a day because it’s part of ablution and it has to be done before every prayer, and this represented our confounding variable.

## Conclusion

Our community is a vital part of our identity; this statement correlates with all the results obtained. It showed high compliance rates, starting with 98.8% for compliance to mask use, followed by 93.7% for hygiene use, and 92.2% for social distancing. The most highly adherent social group was highly educated and retired Bahraini females. Hence, the following should be considered as risk factors for maintaining levels of adherence: younger age groups, non-Bahrainis and individuals living in university accommodations, likely due to impatience and social influences. Similarly, age and education level are risk factors to adherence to social distancing. For hygiene use, age and nationality are risk factors. Adherence to mask use was consistent within all demographics throughout the pandemic, driven by both government enforcement in Bahrain and self-protection.

Furthermore, this study demonstrates that laws and apprehension about hurting loved ones or the community were the main reasons why most people took preventive measures. Hence, in implementing any future legislation and rules, the best way to increase compliance is to target cultural and familial aspects. This will increase compliance subconsciously within citizens. This can be done by government campaigns that focus on the emotional influences of COVID-19 and its effect on the community.

## Data Availability

The datasets for this study can be found upon request from the corresponding author.
